# Comparing [^18^F]FDG and [^68^Ga]Ga-FAPI-04 in two canine cases of histiocytic sarcoma

**DOI:** 10.1186/s13550-026-01516-7

**Published:** 2026-09-16

**Authors:** Patricia Beer, Lars Gerchow, Frauke Seehusen, Carlos Vigo, Alain Blanc, Irene Cortinovis, Martin Behe, Roger Schibli, Mirja Christine Nolff, Irene A. Burger

**Affiliations:** 1 Division for Soft Tissue- and Oncologic Surgery of the Clinic for Small Animal Surgery, Vetsuisse Faculty Zurich, University of Zurich, Zurich, Switzerland; 2 Center for Radiopharmaceutical Sciences, PSI Center for Life Sciences, Villigen, Switzerland; 3 Institute of Veterinary Pathology, Vetsuisse Faculty Zurich, University of Zurich, Zurich, Switzerland; 4 Department of Physics, ETH Zurich, Zurich, Switzerland; 5 Department of Chemistry and Applied Biosciences, ETH Zurich, Zurich, Switzerland; 6 Department of Nuclear Medicine, Kantonsspital Baden, affiliated Hospital for Research and Teaching of the Faculty of Medicine of the University of Zurich, Baden, Switzerland; 7 Department of Nuclear Medicine, University Hospital Zurich, University of Zurich, Zurich, Switzerland; 8 Department of Health Sciences and Technology, ETH Zurich, Zurich, Switzerland

**Keywords:** Imaging of ultra-rare sarcoma subtypes, Upstaging, Theranostics, Animal model, Comparative oncology

## Introduction

During the past 10 years, the use of Fibroblast Activation Protein Inhibitors (FAPI) has gained increased interest for PET imaging and theragnostic approaches in human sarcoma patients [1–6]. The currently available literature is controversial with respect to the impact of FAPI PET regarding up-staging of sarcoma patients compared to [^18^F]FDG, mainly due to subtype dependent variations in enhancement behavior [2–8]. Histopathology analysis showed, that over 70% have high accumulation in stromal cells and 50% of sarcomas also show intense cellular uptake [9].

Reported advantages include the option to include characterization of tumor heterogeneity, monitor and predict treatment response and to select cases suitable for radioligand therapy [2, 10, 11]. First reports utilizing FAP targeting for radioligand therapy in progressive sarcoma patients have recently been published, reporting favorable initial results [5, 10–14]. Further studies investigating the impact of this therapeutic option are currently underway.

However, FAPI accumulation is subtype dependent, and data regarding FAP expression is lacking for some rare sarcoma subtypes. Among these is histiocytic sarcoma (HS). In humans HS is an ultra-rare malignancy that belongs to the group of malignant histiocytic neoplasms, with a reported incidence of approximately 0.17 cases per million individuals [15, 16]. In contrary, it is among the most frequent sarcoma subtype in certain selected dog breeds [17–20].

As systematic validation in ultra-rare cancers is challenging due to low case numbers. Naturally occurring disease models - such as predisposed dog breeds - offer a logical translational approach. Due to the described similarities in biologic behavior, abundant presentation due to high case numbers and comparable molecular signatures dogs represent such a model for HS [15, 21, 22]. Our group already showed that FAP expression is very comparable between different soft tissue sarcoma subtypes between humans and dog [23].

To date, data on FAP-expression in canine HS and on the imaging or therapeutic potential of FAPI are completely lacking. Furthermore, there is also no direct comparison between [^18^F]FDG and [^68^Ga]Ga-FAPI-04 PET imaging in this rare tumor subtype for either human or canine patients.

Here, we present the first report of a comparison of [^18^F]FDG with [^68^Ga]Ga-FAPI-04 in naturally occurring HS in two dogs with histopathology work up.

## Materials and methods

### Canine patient characteristics

Two dogs diagnosed with HS and with a clinical indication for a contrast enhanced (ceCT) were included within a prospective study investigating dual isotope imaging in dogs with sarcoma. The study was approved by the veterinary local ethics committee and conducted at the University Hospital Zurich (TVA: ZH59/2024). Written informed consent to enroll the dogs and publish the data was obtained from the owners. The first dog, a 7-year-old female Flat Coated Retriever was presented two weeks after excisional biopsy of a soft tissue mass at the elbow. The mass had recurred locally within two weeks, ceCT was recommended. The second dog, a 10-year-old female spayed Bernese Mountain Dog, had undergone resection of two lung masses that were confirmed to be HS, followed by chemotherapy (Lomustine followed by Vinorelbine due to liver toxicity). Re-Staging 6 months after initiation of chemotherapy was recommended.

### Dual Tracer PET/CT

Scans were performed on a digital time of flight PET/CT scanner (GE Discovery MI Gen1). After a clinical ceCT [^68^Ga]Ga-FAPI-04 was injected with 2 MBq/kg (dog1: 56.9 MBq, dog2: 75.8 MBq) and dynamically scanned for 20 min over the tumor, then a whole-body scan was performed. After this [^18^F]FDG was given with 1.5 MBq/kg (dog1: 48.2 MBq, dog2: 56.9 MBq) dynamic scans were performed over 30 min followed by a whole-body image (dual tracer image).

The isotope-based separation for both tracers investigated within this study, is not part of this preliminary analysis. Here we used the dynamic [^68^Ga]Ga-FAPI-04 PET data to extrapolate FAPI accumulation at the second time point. Based on this a [^18^F]FDG PET only data set was generated by subtraction from the dual tracer image, using custom in-house software (Python 3.12; NumPy 2.3, SciPy 1.16, pydicom 3.0; ETH Zurich, Zurich, Switzerland). Image reconstruction was performed using 3D time-of-flight, ordered subset expectation maximization, 3 iterations and 18 subsets (matrix size of 256 × 256 pixels, axial field of view: 153 mm). A low-dose CT scan was acquired for co-registration with the PET. Lesion analysis was performed based on standardized uptake values (SUV) (activity corrected for body weight and injected dose) maximum, mean and tumor to background ratio using liver parenchyma as background. Image interpretation was done by a dual boarded radiologist and nuclear medicine physician (IAB).

### Histopathology workup

FAP immunohistochemistry (IHC) was performed on 2 μm FFPE sections using a rabbit monoclonal anti-FAP antibody (ab207178, clone EPR20021; Abcam) at a dilution of 1:200. Following heat-induced antigen retrieval in EDTA buffer (pH 9.0), staining was carried out on a Dako Autostainer using the EnVision+ System-HRP Rabbit detection kit (Dako K4003) and DAB chromogen (Dako K3468). Sections were counterstained with hematoxylin. A canine soft tissue sarcoma served as positive control; negative controls were generated by omission of the primary antibody.

## Results

**Fig. 1:**
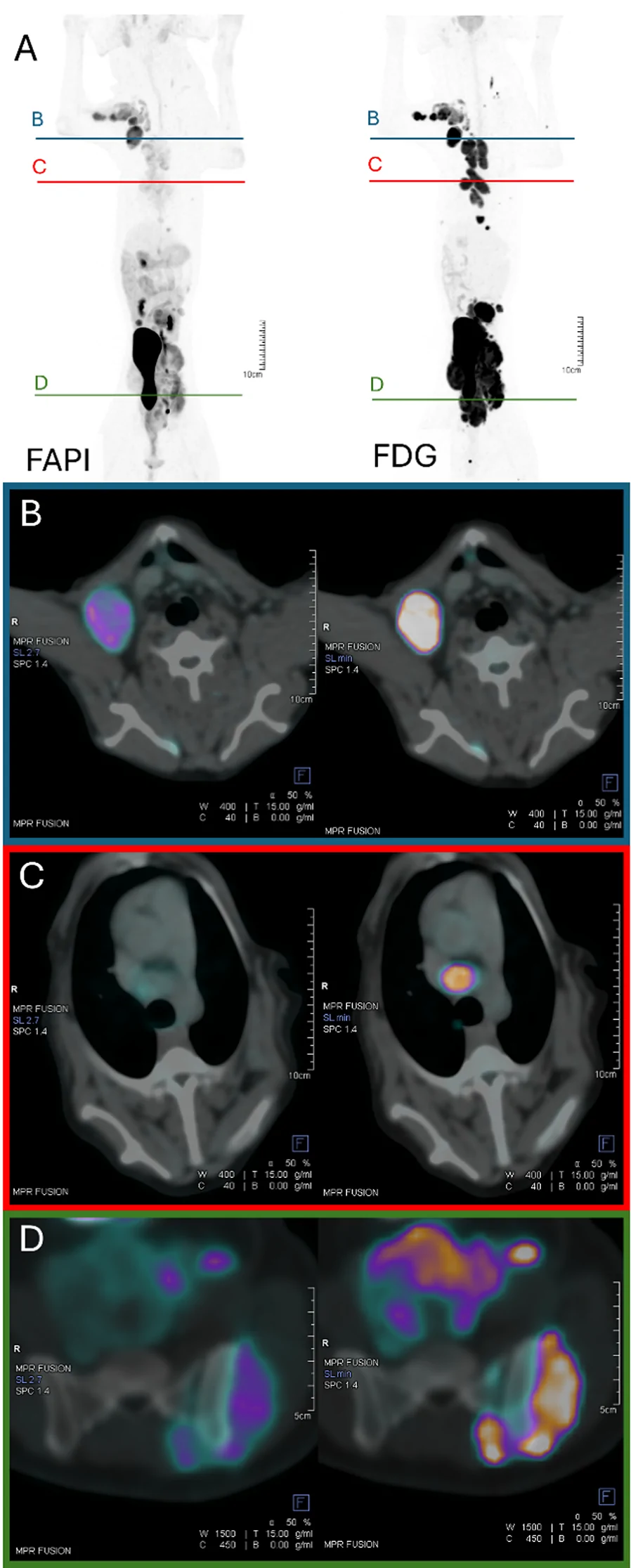
**A**) Coronal maximum intensity projection (MIP) of [^68^Ga]Ga-FAPI-04 PET (left) and extracted [^18^F]FDG PET (right) with extensive, heterogeneous nodal and osseous disease. **B**) Supraclavicular nodal metastasis (blue line) with moderate uptake of [^68^Ga]Ga-FAPI-04, but intense [^18^F]FDG accumulation. **C**) Mediastinal nodal metastasis (red line) with low uptake of [^68^Ga]Ga-FAPI-04, but intense [^18^F]FDG accumulation. **D**) Bone metastasis (green line) with moderate uptake of [^68^Ga]Ga-FAPI-04, but intense [^18^F]FDG accumulation

In dog1, the [^68^Ga]Ga-FAPI-04 PET/CT scan revealed an extensive disease with numerous lymph node metastasis (axillar, supraclavicular, mediastinal, retroperitoneal and iliac). Furthermore, bone and muscle metastasis were detected. The lesions had a very heterogeneous tracer accumulation ranging from low to moderate SUV_max_ on [^68^Ga]Ga-FAPI-04 PET. While axillary lymph nodes had a moderate tracer accumulation, more distant lesions were only minimally FAPI positive (Table 1; Fig. 1). All lesions showed higher [^18^F]FDG accumulation compared to FAPI uptake. The suspected additional muscular lesions were biopsied, and histiocytic sarcoma was confirmed; anti FAP-IHC confirmed FAP expression in the lesions.

**Table 1:** Lesion quantification

Dog 1	Lesions	FAPI SUV_max_	FAPI SUV_mean_	FAPI Tu/B ratio	FDG SUV_max_	FDG SUV_mean_	FDG Tu/B ratio	FDG+FAPI SUV_max_
	Normal Liver	0.5	0.4		0.9	0.7		
	Axillary lymph node (LN)	15.3	8.3	30.6	22	14.5	24.4	28.9
	Mediastinal LN	3.3	1.95	6.6	15.6	10.8	17.3	17.5
	Retroperitoneal LN	5.1	2.6	10.2	17	11.5	18.9	19.2
	Sacrum	10	6.15	20	21	12.9	23.3	26.7
	Thoracic vertebra	2.8	1.5	5.6	18.5	5	20.6	20.8
	Muscle metastasis 1	5.6	3.6	11.2	15.9	10.9	17.7	19.4
	Muscle metastasis 2	8.8	5.5	17.6	18.5	13.2	20.6	24
Dog 2	Normal Liver	4.2	3		5	4.6		
	Lung metastasis	1.7	1.2	0.4	5.9	2.9	1.2	7.5
	Retroperitoneal LN	7.9	3.5	1.9	7.3	3.2	1.5	13.9
	Subcutaneous granuloma	12.4	9.3	3.0	19.5	12.5	3.9	22.3

SUV_max_ (Maximum Standardized uptake value) and mean SUV (SUVmean) as well as the SUV_max_ tumor to background (liver) ratio (Tu/B ratio) on scan 1 after 25 min for [^68^Ga]Ga-FAPI-04 and on scan 2 after 60 min for [^18^F]FDG + [^68^Ga]Ga-FAPI-04. SUV_max_ and SUV_mean_ from the virtually subtracted [^18^F]FDG scan, based on kinetic modeling

**Fig. 2:**
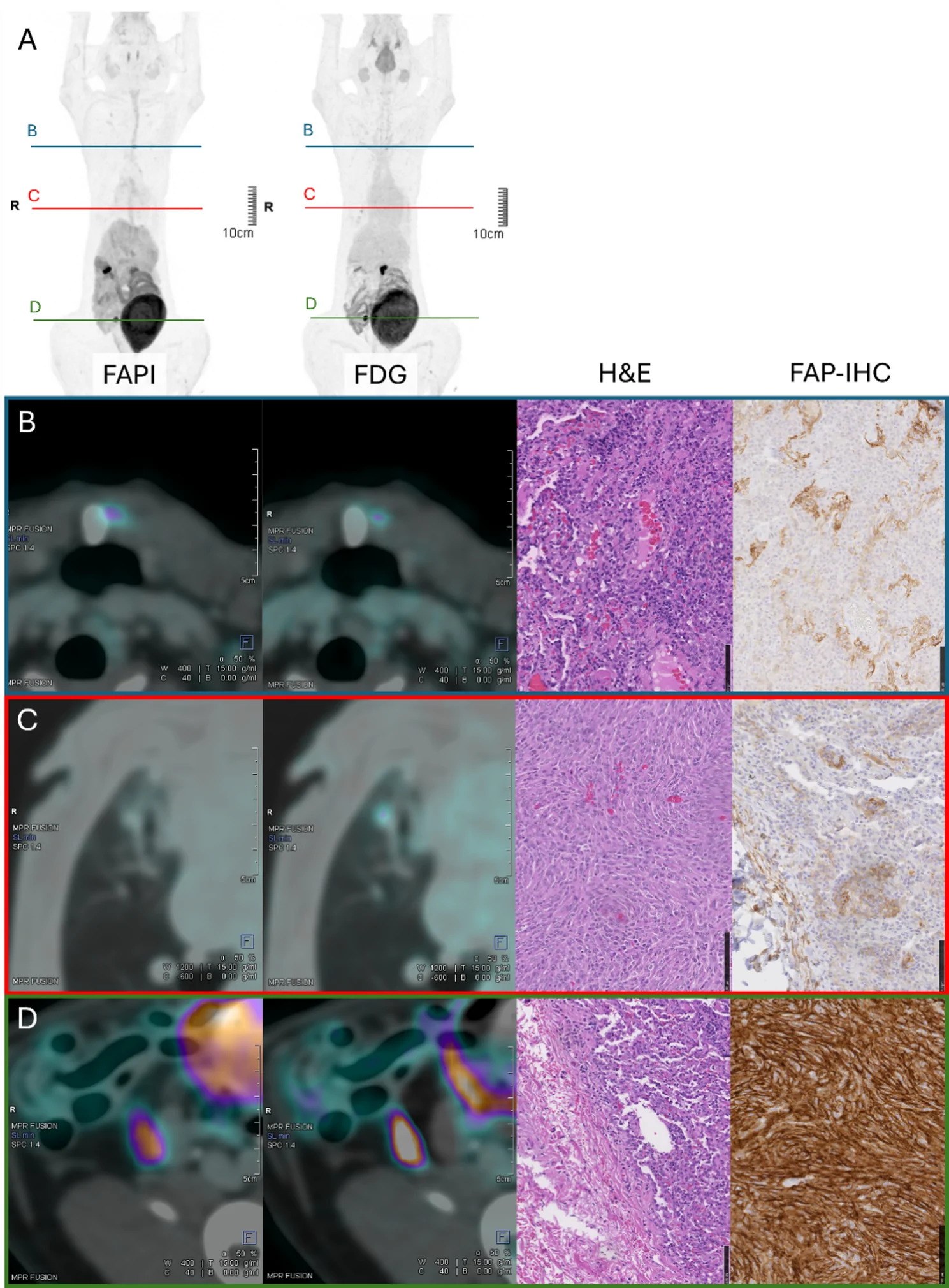
**A**) Coronal MIP of [^68^Ga]Ga-FAPI04 PET (1st colon) and extracted [^18^F]FDG PET (2nd colon) with three heterogeneous lesions. **B**) Subcutaneous, benign granuloma (blue line) with moderate uptake of [^68^Ga]Ga-FAPI-04, as well as [^18^F]FDG accumulation, with high number of lymphocytes on Hematoxylin and Eosin (H&E) staining (3rd colon) and moderate uptake on FAP-IHC (4th colon). **C**) Lung metastasis (red line) with low uptake of [^68^Ga]Ga-FAPI-04, but moderate [^18^F]FDG accumulation, correlating with only mild expression on FAP-IHC **D**) Lymph node metastasis (green line) with high uptake of [^68^Ga]Ga-FAPI-04 and intense [^18^F]FDG accumulation. With intense staining on FAP-IHC

In dog2, [^68^Ga]Ga-FAPI-04 PET revealed three lesions, a subcutaneous nodular lesion at the upper chest with high accumulation, a pulmonary nodule with a diameter of 5 mm, with only faint uptake and a retroperitoneal lymph node metastasis with intense. Histopathology confirmed a lung metastasis with only minimal and a lymph-node metastasis with intense FAP expression. The subcutaneous lesion with intense [^68^Ga]Ga-FAPI-04 accumulation however was a foreign body granuloma due to a blade of grass. (Table 1; Fig. 2).

## Discussion

We describe two cases of [^18^F]FDG and [^68^Ga]Ga-FAPI-04 PET imaging for HS in dogs. Our results show a very heterogeneous FAP expression between different lesions in both cases and an overall superior performance of [^18^F]FDG for lesion detection in this highly aggressive sarcoma subtype. This is in line with the only case report on [^18^F]FDG for HS in human reporting intense accumulation [24]. Until now there are only some trials looking into [^68^Ga]Ga-FAPI-04 imaging for sarcoma in human in general, but without HS subtypes [8, 25].

Despite being overall inferior to [^18^F]FDG, some lesions had marked [^68^Ga]Ga-FAPI-04 accumulation, while the overall background was very low. Given that in humans HS are characterized by an exceptionally aggressive biological behavior, poor treatment response and consequently high mortality rates [15, 16, 21]. The grave prognosis, with a median overall survival of approximately 6 months, and virtually all patients succumbing to the disease, emphasizes the need of novel therapy options [15, 16, 21]. Therefore, FAPI might still be a potential theranostic target for some HS patients.

Given that ceCT is a standard for tumor evaluation in canine HS and needs full anesthesia, the direct combination of two sequential PET scans after a clinical ceCT, is only slightly prolonging the intervention, without any additional stress or risk for the dog. We therefore established a collaboration between a nuclear medicine department and the veterinary clinic, to offer ceCT with dual tracer PET to canine sarcoma patients. We are convinced that, albeit these results have been obtained in dogs, the information is highly relevant in a comparative context, based on the documented substantial overlaps in clinical behavior and molecular features of sarcomas between dogs and humans [23]. The heterogeneity in tumor uptake in both dogs further emphasizes that sarcoma cannot be fully understood with small tumor samples and single biopsies. Both cases demonstrate that a single tissue biopsy from any leison would not have been representative for the entire tumor burden. At this point, we cannot correlate this finding to a reliably underlying mechanism. The fact that the newly developed metastatic lesion in dog 2 showed less FAP expression than the primary is concerning, as a lower FAP expression in fast growing lesions might be a limiting factor for subsequent therapy. However, further investigation of this phenomenon and the overall inter- and intratumor FAP heterogeneity is needed. We believe that canine HS can be instrumental to validate future theranostic targets.

## Conclusion

Based on this preliminary data, for the rare subtype of HS, the heterogenous FAP expression could limit the sensitivity for [^68^Ga]Ga-FAPI-04 PET compared to [^18^F]FDG PET imaging for diagnosis. However, in selected cases FAP might still be a valuable theranostic target. These initial findings warrant further investigation.

## Supplementary Information

Below is the link to the electronic supplementary material.

Supplementary Material 1.

## Data Availability

Data are available upon reasonable request to the corresponding author.

## Abbreviations

ceCT: Contrast enhanced CT
FAPI: Fibroblast Activation Protein Inhibitors
FDG: Fluorodeoxyglucose
HS: Histiocytic Sarcoma
IHC: Immunohistochemestry
SUV: Standardized Uptake Values
TVA: Tierversuchsantrag
