# Supplementary material for: Comparing [18F]FDG and [68Ga]Ga-FAPI-04 in two canine cases of histiocytic sarcoma
Source: EJNMMI Res. 2026 Sep 16;16:155. doi: 10.1186/s13550-026-01516-7 (PMC13743648; doi:10.1186/s13550-026-01516-7)
Supplement: Supplementary file 1 [file 13550_2026_1516_MOESM1_ESM.docx]

Reviewer #1: The short communication titled "Comparing [¹⁸F]FDG and [⁶⁸Ga]Ga-FAPI-04 in two canine cases of histiocytic sarcoma" by Beer and colleagues is about important research results addressing the timely topic of FAP expression in sarcoma. The authors used a canine model of a rare sarcoma subtype. The animal model and study topic are interesting, and the results fit the scope of the EJNMMI Research journal.

A 1.0: thank you very much for this kind comment

The manuscript is well-written. Some minor corrections are listed below that the authors should address when improving their manuscript.

1. Page 2, line 37: "Data regarding FAP expression is lacking for some rare sarcoma subtypes": To better guide the reader, it would be helpful to explain that FAP expression in sarcoma can be detected in tumor cells (in at least some types of sarcoma), not just in cancer-associated fibroblasts. This important information should be included in the introduction, along with the appropriate supporting literature, as it explains why FAPI uptake can be used for sarcoma staging.

A 1.1: We agree and added a sentence tot he introduction. “*Histopathology analysis showed, that over 70% have high accumulation in stromal cells and 50% of sarcomas also show intense cellular uptake.”*

2. Page 2, line 54: "Patient characteristics". Please change the headline to "Canine model".

A 1.2: Well, this are real patients and not artificial tumor models, but we understand that in a more human oriented journal this can be misleading – we would therefore suggest: “Canine patient characteristics” and hope this is ok.

3. Page 3, line 69: "canned". Should be "scanned".

A 1.3: Thank you – corrected.

4. Page 4, line 74: "generated by subtraction". Please add the software (with version and company) used for the image substraction procedure.

A 1.4: added: using custom in-house software (Python 3.12; NumPy 2.3, SciPy 1.16, pydicom 3.0; ETH Zurich, Zurich, Switzerland)

5. Page 4, line 79: "done". Should be "was done".

A 1.5: Thank you – corrected.

6. Page 4, line 88/89: "anti FAP immunohistochemistry (IHC)". Please add to the Material and methods section a brief description of the method, including the source of all antibodies used and their dilutions.
A 1.6: This was added to page 4.

7. Page 5, line 106: "resultingly". Sounds awkward, please rephrase and use "with consequently" or "resultant".
A 1.7: This was changed to consequently.

8. Page 5, line 109: "FAPI might still be a potential target". Please rephrase - do the authors mean "FAP-mediated radiotherapy might still be an alternative option"?
A 1.8: Yes – we added that we see FAP as still potential theranostic target, in some patients.

9. Page 5, line 119: a comma is missing after "however".
A 1.9: Thank you – corrected.

Reviewer #2: The manuscript is clearly organized, the methodology is appropriate, and the histopathological correlation adds significant value to the imaging findings. The work provides novel preliminary data that may stimulate further translational research on FAPI imaging and theranostics in histiocytic sarcoma.

A2.0: Thank you

Authors should address the absence of an abstract, strengthen the discussion of the translational implications, and moderate some of the conclusions given the limited sample size:

A2.1: Abstract: we had an abstract in the manuscript but were told by the journal to remove it: *Histiocytic sarcoma (HS) is a rare very aggressive sarcoma subtype in humans, with limited data on imaging and therapy options, but common in some canine breeds. This allows testing different imaging targets, such as the Fibroblast Activation Protein Inhibitor ([^68^Ga]Ga-FAPI-04) in a realistic tumor model. Two dogs with HS were scanned within a collaboration between the veterinary clinic and the nuclear medicine department. Both dogs underwent a clinically indicated CT and a dynamic [^68^Ga]Ga-FAPI-04 scan, followed by an [^18^F]FDG injection. PET images were compared to FAP immunohistochemistry (IHC). Both dogs had multiple HS metastasis with overall higher [^18^F]FDG accumulation compared to [^68^Ga]Ga-FAPI-04 uptake. There was a good correlation between FAP-IHC and [^68^Ga]Ga-FAPI-04 in all lesions with available histopathology. Our cases show that for the rare subtype of HS, the wide variability in FAP expression, might limit the sensitivity for [^68^Ga]Ga-FAPI-04 PET, while all lesions had very intense [^18^F]FDG accumulation.*

Translational significance should be strengthened:
The introduction appropriately highlights the similarities between canine and human histiocytic sarcoma. However, the Discussion could better emphasize how naturally occurring canine disease can serve as a valuable model for evaluating novel PET tracers and future theranostic strategies in humans
A2.2: We agree and added a sentence to the discussion.

Conclusions should be more cautious
Based on only two cases, the statement that [18F]FDG outperforms [68Ga]Ga-FAPI-04 should be tempered. The manuscript should emphasize that these are preliminary observations that require validation in larger cohorts.
A2.3: we agree and used changed the conclusion accordingly.

Quantitative analysis
Although the small sample size limits statistical analysis, a more comprehensive presentation of quantitative PET parameters (SUVmax, SUVmean, lesion-to-background ratios) for all lesions would improve the scientific value of the paper.
A2.4: We added the SUVmean, and a tumor to liver ratio to the table.

Discussion of heterogeneous FAP expression
The heterogeneous FAP uptake observed among different metastatic lesions is one of the most interesting findings of the study. The authors should expand the discussion on the possible biological mechanisms underlying this heterogeneity and its implications for patient selection for FAPI-based radioligand therapy.

A2.5: We agree and added a sentence to the discussion.
